# Antiseptic Surgery

**Published:** 1880-07

**Authors:** T. S. Floyd

**Affiliations:** Sedgwick, Kansas


### ANTISEPTIC SURGERY.

BY T. S. FLOYD, M.D., SEDGWICK, KANSAS.

In the domain of antiseptic surgery there have been during the past year no special discoveries or improvements, either in the agents to be used or their mode of application. In England and Scotland, especially, the full method of Lister has had its advocates and its opponents, and the controversy between them has, in some instanses, been quite bitter, and as yet without decisive results. One class claiming that equally good results were obtained under the old method where sufficient care and cleanliness were observed, as under the new, a statement which Mr. Spence, of Edinburgh, has sought to demonstrate in a series of cases drawn from the records of the Edinburgh Infirmary.

It seems to me that our practice must be largely influenced by the views we take as to whether the septic and deleterious influences and agents we have to combat, as surgeons, come from within, or are received from without, or from both sources, or either.

No one who is familiar with the experiments and researches of Pasteur, and embodied in his work on the "Organized Corpuscles Existing in the Atmosphere," or with the experiments of Prof. Tyndall and his replies to Dr. Bastian on the question of spontaneous generation, or with the labors of Prof. Cohn, of Breglau, who says, "No putrefaction can occur in a nitrogenous substance if its bacteria be destroyed and new ones prevented from entering it. Putrefaction begins as soon as bacteria, even in the smallest numbers, are admitted either purposely or accidentally. Its progress is in direct proportion to the multiplication of the bacteria, it is retarded when they exnibit a low vitality, and is stopped by all influences which either hinder their development or kill them. All bactericidal media are therefore antiseptic and disinfecting," who can doubt that many septic agents may be received upon open wounds or abraded surfaces from the atmosphere itself as well as from contaminated dressings or instruments, and that, therefore, any or all agents which have power to destroy or to prevent the access of these germs must be more or less useful, if not altogether indispensable in surgical practice?

But I am not of those who look for no other source of poisoning than bacteria, and no other medium of contamination than the atmosphere. While an almost unanimous verdict has been accorded the antiseptic method of treatment, it by no means follows that all the details of Mr. Lister's plan must be followed to obtain any useful results. Some good has already resulted from the discussion of his method, as it bas prompted a closer inquiry into the therapeutic action of the agents heretofore in use, and a search for new, and in some cases, perhaps, more useful and expedient ones; it has also raised the question whether other and less troublesome, cumbrous and expensive methods than Lister's would not be equally serviceable. The carefully devised experiments of Prof. Lewis A. Stimson, of New York, seem to demonstrate that carbolic acid spray, as usually employed, is not effective to purify, and does not destroy the putrefactive germs floating in the air, and that all the benefits of the acid can as well be obtained by the solution or localized spray directly applied to the wound.

The beneficial effects of carbolic acid dressings no one will probably deny, but whether these are to be more directly imputed to its bactericidal power than to its powerful sedative action is still an open qdestion.

Permit me now to ask your attention to an article which, although it has received high commendation from distinguished sources, is not in so common use as I believe its merits would entitle it. I refer to the eucalyptus globulus. I will not weary you with its botanical history, as that can be found in most of the recent text books on materia medica. Something over two years ago my attention was drawn to the probable value of this agent as a surgical dressing, and my first application was of the fluid extract in the following case: A boy about 12 years of age came into my office his hand covered with blood, and told me his fingers had been caught between a rope carrying a heavy weight and the edge of an iron pully over which it ran; an examination showed the terminal phalangers of the first and second fingers to be severely crushed, and the joint of the second finger laid open. Believing that amputation would be necessary, and as his parents were not present, I straightened the crushed fingers on a slip of pasteboard and confined them with a few turns of a roller open at the ends, and then saturated the fingers and bandages with the fluid extract of eucalyptus, and sent the boy home, saying I would call and see him later. On my visit I found the saturated bandage almost as hard as a plaster splint; and the fingers giving no pain, as the bandage was open at the ends so that I could easily watch for any change that might demand interference, I resolved to allow the dressing to remain and apply through the open ends the fluid extract of eucalyptus, one part to seven of water. As neither pain or suppuration supervened, this dressing was allowod to remain for ten days, when I removed it and found the fingers I had first expected to remove, nicely healed; the joint was stiff and the nails gone, but the latter have since grown out and the icint, under passive motion, recovered almost its natural mobility. I have described this case at length, as it illustrates the method I have employed in a large number of injuries to fingers and hands, always allowing the first bandage, when it could be neatly and closely applied, to remain until the wounds were healed, and in each case the result has been entirely satisfactory.

In cases similar to the one described it is especially applicable, as the saturated dressing hardens and forms a sufficient support.

So satisfactory thus far had been my experience with this agent in minor surgery that I resolved to extend its application to more serious cases, and did not wait long, for an opportunity presented itself in the case of a middle aged German who had received a compound comminuted fracture of the left forearm by its being crushed in the geering of a wind-mill, of so serious a nature as to demand amputation at about the junction of the upper and middle thirds, which was done on the 9th day of April 1879. Sponges and bandages soaked in a twenty per cent. solution of carbolic acid in water, and instruments washed in same, sutures and adhesive stamps applied in the usual manner. The stump was enveloped in several folds of the thin muslin, commonly called cheese cloth, and kept saturated with the fluid extract of eucalyptus, one part to seven of water, this was the only dressing ; there was very little pain or suppuration, and on the 28th, nineteen days after the operation, the patient was discharged, the stump healed nicely and needed no further surgical care.

Since that time I have used eucalyptus in some form in nearly all my surgical cases with equally good results.

In October, 1879, I requested Parke, Davis & Co., of Detroit, to furnish me, if they could, a fluid preparation of eucalyptus devoid of resin only, to which request they kindly complied by sending me four preparations. Numbers one and two containing all the basic, neutral and acid principles, in various proportions; number three all the ingredients of the leaves in the same proportion as in the fluid extract except the resin, and number four was the oil. The first three are readily miscible with water, and can be used as a spray or as a simple dressing. The oil is readily soluble in an equal part of strong alcohol, and will mix with oil or parafine; it is devoid of toxic action and is powerfully antiseptic. Lint, gauze, or muslin saturated with the prepared oil makes an elegant and convenient dressing, and agreeable in smell; it destroys all fœtid odors without substituting another offensive one.

In October last, having these various preparations to use, samples of which I show you to-day, I instituted the following experiments to test the power of each to prevent putrefaction :

I prepared a strong infusion from a parcel of musty hay, and selecting five clean bottles, placed in each of them three ounces of this infusion. To the first three I added one drachm, respectively, of Nos. one, two, and three, to the fourth, one drachm of the ordinary fluid extract, and to the fifth, twenty minims of the oil dissolved in forty minims of alcohol, so as to make a drachm. These were all left open in a warm place in my office. In ten days the bottles containing the first three, or extracts without resin, showed signs of decay, but there was no change in the two containing the common fluid extract or the oil. All these bottles remained open until cold weather, when I corked them and put them away in saw-dust to prevent freezing. A month ago, or about six months from their preparation, I again opened them, the non-resinous preparations were quite fœtid, but no putrefaction had yet taken place in those treated with the oil or resinous extract. At this date they were still standing open in my office perfectly sound.

Does not any agent possessing such powerful anti-putrefactive power with absolute freedom from poisonous qualities deserve a more extended use and trial?

